# Determination of the Entire Existence Composition Range of CrMnFeCoNi High-Entropy Alloys Using Sintered Diffusion Multiple Method

**DOI:** 10.3390/ma18020295

**Published:** 2025-01-10

**Authors:** Ryuta Yurishima, Ayako Ikeda, Teruyuki Ikeda

**Affiliations:** 1 Graduate School of Science and Engineering, Ibaraki University, 4-12-1 Nakanarusawa, Hitachi 316-8511, Ibaraki, Japan; 2 Research Center for Structural Materials, National Institute for Materials Science, 1-2-1 Sengen, Tsukuba 305-0047, Ibaraki, Japan; ikeda.ayako@nims.go.jp

**Keywords:** high-entropy alloys, phase diagram, sintered diffusion multiple method

## Abstract

The sintered diffusion multiple (SDM) method, which has been developed in our research group, has been applied to determine the entire composition range of the CrMnFeCoNi high-entropy alloy stereoscopically and continuously over nearly the entire range. The samples were prepared by sintering mixed elemental powders and were annealed at 970 °C or 800 °C. Several hundreds of thousands of points were analyzed at random within the samples for chemical compositions using electron probe microanalysis. With the assumption that ideally, only chemical compositions of existing phases at the temperature of annealing are obtained, the compositional data thus obtained were analyzed to estimate the phase boundaries of the high-entropy phase, including the Cantor alloy composition, assuming local equilibrium within the samples. The analysis includes the determination of point densities and their slopes in the space of chemical composition. The results are shown in the tetrahedral compositional space, with vertices for the Cr, Mn, and Fe atomic fractions and the sum of the Co and Ni fractions. One of the features found in this work is that the high-entropy phase exhibits a wide compositional range in the Fe-CrMnCoNi direction. The estimated phase boundary compositions are found to be in good agreement, within an error range 3 at.%, with those obtained using samples prepared by the conventional method, where the samples with uniform compositions are equilibrated by annealing, and the compositions of their existing phases are analyzed using EPMA. Thus, the sintered diffusion multiple method is effective in providing an overview of the quinary phase diagrams.

## 1. Introduction

High-entropy alloys (HEAs) have been expected to exhibit unique physical properties since the idea of a high-entropy alloy was introduced in the metallurgy field by Cantor and Yeh [1,2]. HEAs are defined as solid-solution alloys composed of more than five elements with intermediate compositions and hence, stabilized due to extremely high configurational entropies [3]. Such unique atomic arrangements and atomic interactions have the potential to give rise to properties creating the so-called cocktail effect, which is not possible from a simple mixture of elements. The Cantor alloy is well known as a prototype of HEAs, with the FCC structure exhibiting a unique mechanical property, which displays high strength at low temperatures. This FCC phase has been reported to have a wide existence compositional range [4] implying the ability to control the physical properties by changing the composition while retaining the crystal structure. A number of studies examining the stability [5,6,7,8,9] or the composition range [10,11,12,13,14,15,16,17,18,19,20] of the HEA phase have been conducted experimentally and with the aid of CALPHAD calculations, even for the Cr–Mn–Fe–Co–Ni system alone. However, all these studies have been conducted on pseudo-binary sections of the quinary system and hence, the entire picture of the existence range of the high-entropy Cr–Mn–Fe–Co–Ni alloys is still unknown. A tremendously vast compositional space of a multicomponent system such as HEAs requires an enormous amount of effort to examine their detailed phase diagrams.

The novel material exploration, aided by a variety of calculations and data science, has thus been developed. To achieve high precision results using these approaches, a large amount of experimental data is essential. Regarding experimental techniques for creating an equilibrium phase diagram, a conventional method is often used, where samples with basically homogeneous compositions are subject to long-term annealing at constant temperatures for equilibration, and one set of data on one tie line per sample is obtained [21]. However, for multicomponent alloy systems, too many experiments are required to complete a system. To accelerate data acquisition, therefore, methods using diffusion multiples [22,23] are available. It is, however, not necessarily easy to prepare samples for this method, since it requires bonding multiple times and may cause interfacial debonding. In addition, the number of constituent elements of the alloy system that can be examined using the diffusion multiples technique is limited geometrically up to four; it cannot be applied to multicomponent systems with more than five components.

Recently, as another option, our group has lately developed a sintered diffusion multiple method [24,25], in which a mixed powder of multiple elements is sintered. A sample prepared by this method can be considered to be an assemblage of micro diffusion couples, triplets, quadruplets, and so on. Assuming local equilibrium in an annealed sample, it is possible to obtain information on the equilibrium phase diagram in a wide compositional space from a single sample. The advantage of this method is that the sample preparation is simple, and the number of constituent elements of the alloy under examination is not limited up to four for geometric reasons. The experimental data for the Al–Fe–Si [24,25] and Ni–Co–Ti–Al system [26] obtained by this method in previous studies are in good agreement with the reported phase diagrams.

In this study, the sintered diffusion multiple method is applied to the quinary phase diagram of the Cr–Mn–Fe–Co–Ni system. This work is the first trial to apply the method to a quinary system. We determine the compositional range of the high entropy FCC phase, the so-called Cantor alloy, stereoscopically, and discuss the usefulness of the sintered diffusion multiple method to examine the phase diagrams of multicomponent systems.

## 2. Materials and Methods

To prepare the SDM samples, chromium powder (99%, 63 μm pass), manganese powder (99.9%, 300 μm pass), iron powder (99%, 150 μm pass), cobalt powder (99.9%, 150 μm pass), and nickel powder (99.9%, 150 μm pass), all of which were from Kojundo Chemical Laboratory Co., Ltd. (Tokyo, Japan), were mixed at a molar ratio of 1:1:1:1:1 and sintered in a graphite die with a 12 mm diameter at 700 °C to 800 °C for 5 to 10 min at a pressure of 11.3–25.5 MPa. The SDM samples were annealed at 800 °C for 256 h or 970 °C for 1 h to 256 h to introduce compositional gradients among multiple chemical elements and were water-cooled. To demonstrate, using a standard case of the SDM method, the temperature needs to be chosen within the range at which the alloy system is composed of only solid phases at this temperature. On the other hand, larger diffusivities are preferred in terms of the time required for the experiments. From these two points of view, 970 °C was chosen as the temperature to be examined. In addition, 800 °C was chosen because at temperatures around 800 °C, phase stability has been observed in the literature [6,7,8,9], and hence, it is important to investigate the phase equilibria at 800 °C. We actually began the experiments with annealing at 970 °C, without knowledge regarding the diffusion depth that would be obtained. That is why we performed the annealing for various periods. After these experiments, we expected that an annealing for 256 h would be enough to obtain information on phase equilibria of the high entropy phase at 800 °C in nearly the entire range. This is the reason why we conducted annealing just for 256 h at 800 °C, while annealing was performed for various periods at 970 °C.

**Figure 1:**
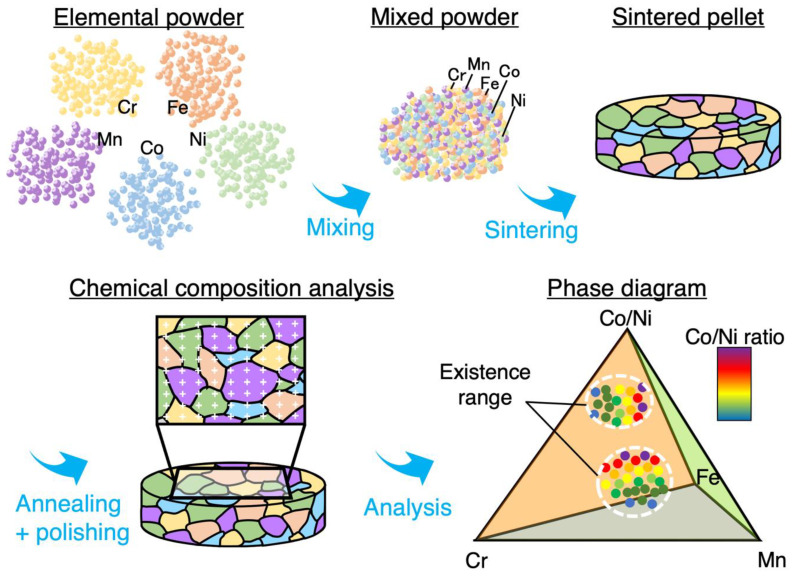
Schematic view of sintered diffusion multiple method.

The samples were cut into halves and embedded in conductive resin, and the cut surfaces were polished for observation using abrasives #320, #400, and #600, followed by polishing with a series of diamond slurries with particle sizes of 9 μm, 3 μm, and 0.05 μm, respectively. A schematic view of the experiment is shown in Figure 1.

The microstructures of the SDM samples were observed using a scanning electron microscope, equipped with an electron-probe microanalyzer with wavelength dispersive X-ray spectroscopy (EPMA; EPMA-8050G, Shimadzu Corp., Kyoto, Japan). The chemical compositions were mapped in a grid pattern at 1–2 μm intervals for several regions with 200 μm × 200 μm areas at an acceleration voltage of 15 kV and a 1 μA current for 8 ms per point. The measured intensities of the characteristic X-rays Cr Kα, Mn Kα, Fe Kα, Co Kα, and Ni Kα were converted to chemical compositions via the ZAF-correction method using intensities from elemental standard samples.

In order to examine the validity of the phase diagram determined using the SDM samples, it was compared with the phase equilibria determined by a conventional method, where two-phase samples with various average compositions are prepared and annealed for a long period of time for equilibration, and the compositions of the constituent phases are analyzed. For this purpose, the samples were synthesized by arc melting under an argon atmosphere. The starting materials are chromium chip (99.87%, Tosoh Corp., Shunan, Japan), manganese grain (99.9%, Kojundo Chemical Laboratory Co., Ltd., Itado, Saitama, Japan), iron grain (99.99%, Kojundo Chemical Laboratory Co., Ltd., Itado, Saitama, Japan), cobalt flake (99.92%, Xstrata plc, Zug, Switzerland), and nickel pellets (99.80%, vale, Toronto, ON, Canada). Heat treatments for homogenization and equilibration were performed in a quartz tube sealed in a vacuum at less than 2 Pa. The temperature was maintained at 1000 °C for 48 h or at 1150 °C for 12 h for homogenization and then lowered to 970 °C at 1 K/min, held for 200 h for equilibration, and then quenched in water. Another set of samples was held at 800 °C for 400 h for equilibration after homogenization. The specimens were then cut and embedded in conductive resin. The surfaces were then polished in the same manner as the SDM samples. The phase boundary compositions were obtained by EPMA with a point analysis mode at an acceleration voltage of 15 kV and a current value of 20 nA for 1 min.

## 3. Results and Discussion

### 3.1. Display of Raw Chemical Composition Data in the CrMnFeCoNi SDM Samples

This is the first study in which the SDM technique is applied to a quinary system, and so the methods for the construction of phase diagrams and displaying the analytical results have not yet been established. The phase diagrams for the multicomponent systems are drawn with geometric figures, with each pure element as an end component; examples include a line for a binary system, a triangle for a ternary system, and a tetrahedron for a quaternary system. In this work, a tetrahedron is used to display the phase diagrams for the quinary Cr–Mn–Fe–Co–Ni system. Since a tetrahedron has four vertexes and is less than the number of component, one vertex is used for the sum of fractions of two elements, and the ratio between them is shown using a color scale. We chose Co and Ni for the two elements because they form a proportional solid solution, and hence, their elemental characters are similar to each other. Thus, the equiatomic composition CrMnFeCoNi is located at (*x*_Cr_, *x*_Mn_, *x*_Fe_, *x*_Co/Ni_) = (0.2, 0.2, 0.2, 0.4) in the compositional tetrahedrons used in the following displays.

**Figure 2:**
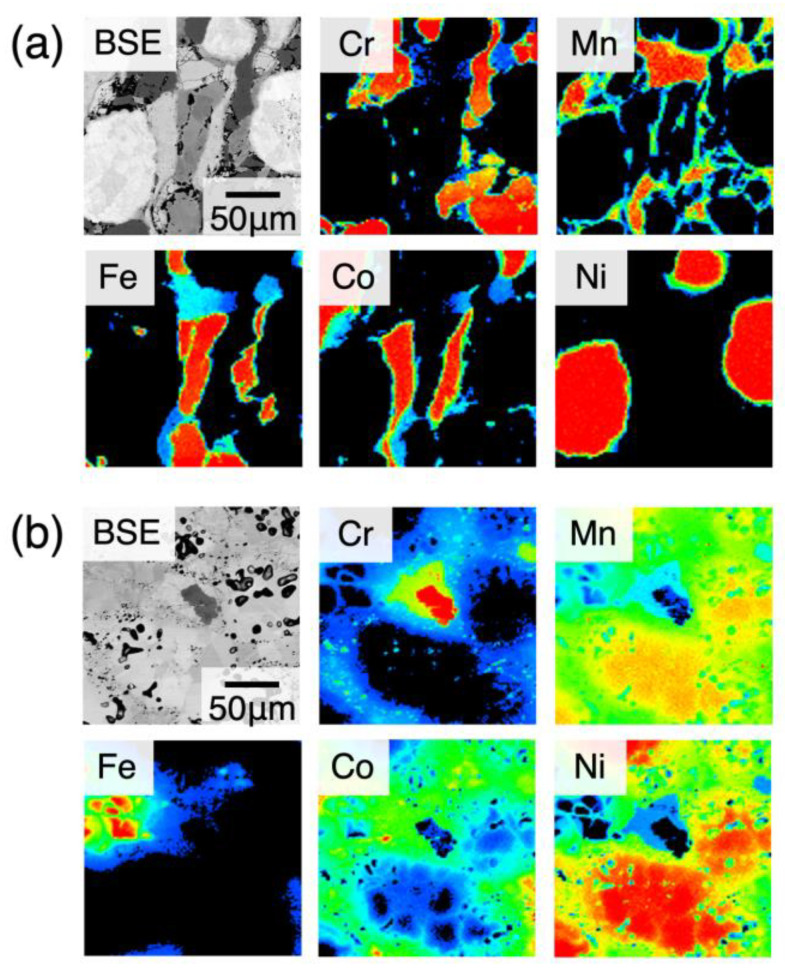
Microstructures in the as-sintered state (**a**) and after annealing at 800 °C for 256 h (**b**). The monochrome images are backscattered electron images, and colored images are characteristic X-ray maps for the respective elements.

The raw chemical composition data of 920,000 points at 970 °C and 720,000 points at 800 °C were obtained from SDM sample diffusion annealed at 970 °C for 1, 4, 16, 64, and 256 h and 800 °C for 256 h. The microstructures obtained by a scanning electron microscope are shown in Figure 2. From Figure 2a, tens of micrometers diameter or larger particles of each element (Cr, Mn, Fe, Co, Ni) are recognized. Composition mixing due to interdiffusion is limited in very small ranges in the as-sintered sample. Thus, we succeeded in preparing a sample of a sintered diffusion multiple. At this stage, in most regions, each element still remains as an element. Figure 2b reflects the microstructure after interdiffusion proceeded to larger ranges after the 256 h annealing, where the interfaces between different particles of different elements are indistinct because of interdiffusion. Thus, annealing after 256 h yields considerable information on the phase equilibria of this multicomponent system. In Figure 2b, one can recognize tiny dots, which are not considered to reflect real compositional variations, since a typical X-ray intensity in the current experiments, around 800 counts per pixel, gives an error of ±30 counts (~3.5%) and provides the contrast in the mapping images.

**Figure 3:**
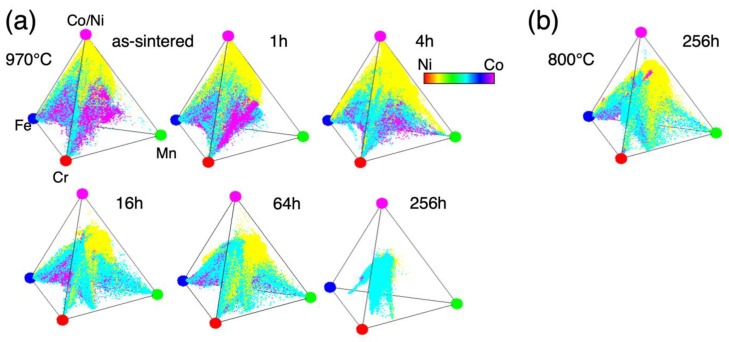
Tetrahedral plots of compositions measured from the Cr–Mn–Fe–Co–Ni sintered diffusion multiple samples annealed at 970 °C (**a**) and 800 °C (**b**), respectively. The color scale shows the Ni–Co ratio.

The chemical compositions obtained by WDS mapping analysis shown in Figure 2 were plotted in the Cr–Mn–Fe–Co/Ni tetrahedral compositional space of Figure 3. As one can see from the data at 970 °C shown in Figure 3a, as the annealing time increases, the measured compositional points in the sample, on the whole, gradually shift towards the equiatomic composition due to interdiffusion, and come together near the center at the end (~256 h). Therefore, it is reasonable to use data obtained for up to 256 h of annealing. At 800 °C, the only data from the annealing for 256 h seems to cover a large compositional space (Figure 3b). Since it covers a wide compositional range at either temperature, it is hard to identify the compositional range of the phases present merely from Figure 3. Because the compositional analyses are performed at a large number of random points, the measurement points include those obtained from points on phase boundaries in the sample space and provide the apparent compositions in the miscibility gaps in the compositional space.

### 3.2. Analysis to Identify the Compositional Range of Phases

The concept of SDM is to form many micro-diffusion couples, triplets, and quadruplets in a sample in a simple way. Assuming that local equilibrium holds within the samples after diffusion annealing, compositions existing within the sample reflect composition ranges of the existing phases of the equilibrium phase diagrams of the alloy system among the elements used as endmembers of SDM. Based on this idea, compositions within the sample are measured at random by EPMA. However, compositions thus collected involve those in cases where the electron probe happens to be located on the phase boundaries, which are of apparent compositions of mixed phases. Therefore, all measured compositions cannot be regarded as those of the current existing phases as they are.

**Figure 4:**
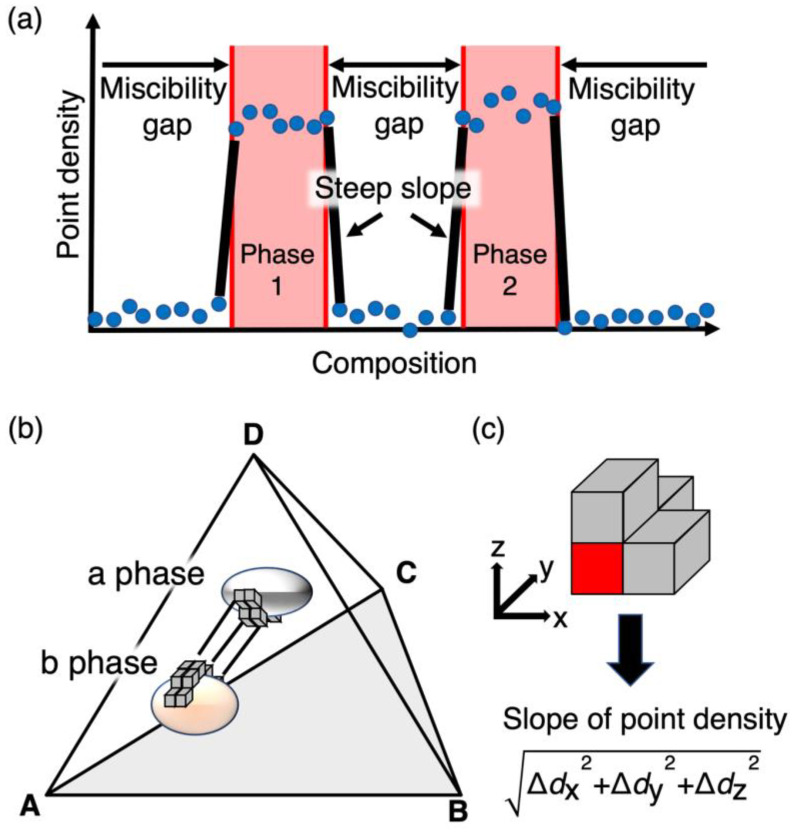
Schematic illustration of the concepts used to analyze composition data obtained from the sintered diffusion multiple samples of the A-B-C-D system: the relationship between measured compositions and point density, the former of which include apparent compositions (**a**), elemental cubes used to determine point density (**b**), and slope of point density (**c**) in the compositional space.

To address this issue, we introduce point density in the compositional space. The tetrahedral compositional space among Cr–Mn–Fe–Co/Ni, Figure 4b, is divided into elemental cubes, with an edge the length of which is one hundredth of an edge of the whole tetrahedral compositional space, and the number of measured compositional points per elemental cube is defined as the point density. As shown Figure 4a, point densities within compositional ranges of existing phases are expected to be higher than those in the miscibility gaps, since measured points are located in miscibility gaps only in cases where the electron probe happens to be located on the phase boundaries. Therefore, the absolute value of the slope of point density is thought to be maximal at the phase boundaries. Thus, the phase boundary surfaces can be represented by the maximal slope of point density.

**Figure 5:**
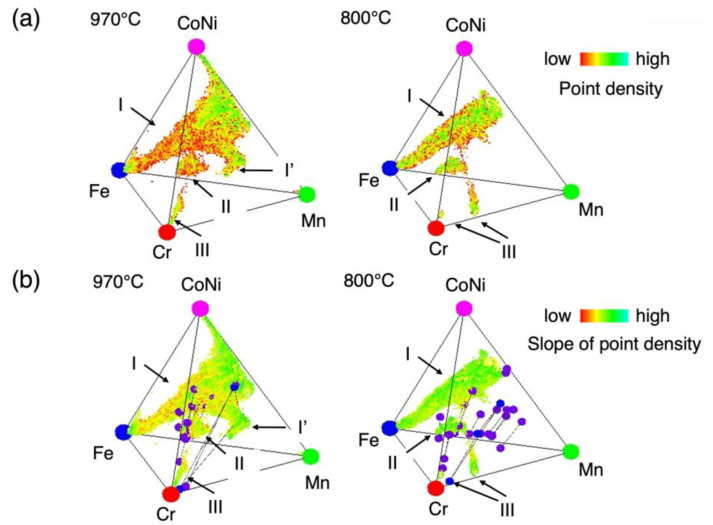
Analysis results of SDM samples showing the point density (**a**) and the slope of point density (**b**) obtained at 970 °C and 800 °C, respectively. In the figure (**b**), tie lines obtained by the conventional method are shown together: segments with purple end points and triangles with blue vertices show two-phase and three-phase equilibria, respectively. The color scales show respective quantities, i.e., point density (**a**) and slope of point density (**b**). The numbers I, II, III, and I′ were labeled to regions where data points gather.

In the practical analysis, the slope of the point density, $\sqrt{∆{d_{x}}^{2}+∆{d_{y}}^{2}+∆{d_{z}}^{2}}$, where Δ*d_i_* (*i* = *x*, *y*, and *z*) is the difference in the point density of the adjacent cubes in the x, y, and z directions, is defined as shown in Figure 4c. A threshold was then set as the lower limit of this point density slope to see the phase boundary surfaces, and the common logarithm of the point density slope was taken between the maximum and the lower limit of the point density slope, displayed using a continuous gradient color scale in Figure 5. The surfaces with colder colors indicate larger slopes of point density and can be considered as a phase boundary surface. The reason for choosing the common logarithm is as follows: experimental point densities are different phase by phase, and hence, the differences between those of the regions within a phase (that is, the existence range of a phase) and outside a phase (the probe of EPMA is located on a phase boundary by chance in the sample space, and hence, the measured points are plotted in-between phases in the compositional space, but their point densities are basically low) could be different phase by phase. In order to highlight the maximal surfaces of the slope of point density, even with low point densities, it should be effective to look at the slope of point density in a logarithm scale. While the information on the Co/Ni ratio is lost, since this analysis was performed within the compositional space of a tetrahedron, with the sum of Co and Ni as one vertex, it can be obtained by referring to the raw chemical composition data.

### 3.3. Composition Ranges of Existing Phases in the Cr–Mn–Fe–Co–Ni System

**Figure 6:**
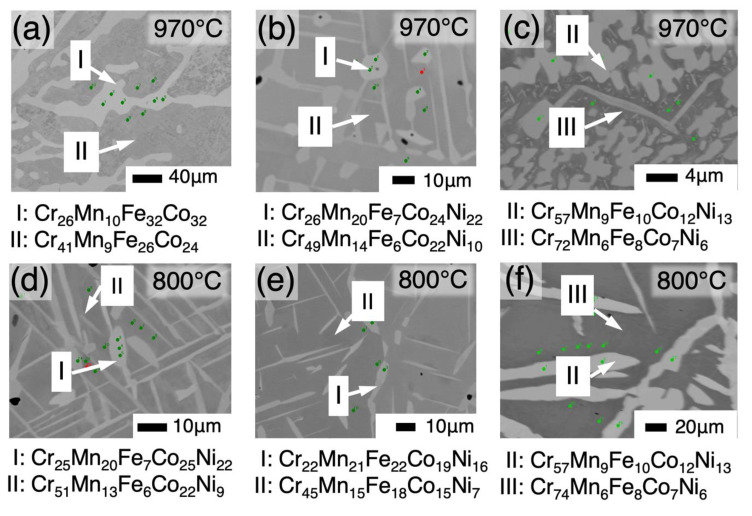
Microstructures observed in samples prepared in the conventional method. The annealing temperature is 970 °C for (**a**–**c**) or 800 °C for (**d**–**f**). The compositions shown below each micrograph are those, in atomic fractions, measured by electron-probe microanalysis at green and red dots for respective phases in the images.

To identify the existing ranges of equilibrium phases in the phase diagrams, we focus on the change in the point density in the compositional space, which is defined as the number of measurement points per elemental cube across the phase boundary; the point density should change rapidly across a phase boundary, as shown Figure 4a. Therefore, the phase boundaries are expected to be represented by the surface with the maximal slope in point density. Figure 5 shows the point density (a) and the slope of point density (b). Supplemental Figures S1 and S2 show the slope of point density from various directions different from those of Figure 5 at 800 °C and 970 °C, respectively. Thus, the phase boundary compositions were estimated stereoscopically and continuously by extracting compositions with a large slope of the point density. To confirm the validity of the estimated phase boundary compositions, we compared them with those obtained using the conventional method. In Figure 5b, the tie lines obtained using the conventional samples are shown, together with the slope of point density. The microstructures of the samples prepared via the conventional method are shown in Figure 6, where the measured compositions of the respective phases in the microstructures are indicated as well. As seen in Figure 6, the size of the microstructure is large enough to determine the compositions of the respective constituent phases by EPMA, in the light of its spatial resolution.

There are three regions recognized in the slope of point density map; the first one is the region which has the largest volume extended to Fe from the Co/Ni–Cr–Mn plane and is labeled as “I” in the figure. The second one, “II”, is the region that is located on the Cr-richer side of region “I”. And then, the last one, “III”, is the region close to the Cr-vertex, which is thought to be the Cr phase.

As found in Figure 5b and Figure S1, in the 970 °C results, many of the phase boundary compositions obtained by the conventional method are located near the maximal slope of the point density surfaces. Figure S3 shows the slope of point density on the cross-sectional plane, including Fe_35_(CoNi)_65_, Mn_43_(CoNi)_57_, Cr_93_Fe_7_, and Cr_90_Mn_10_, together with the phase boundary compositions determined by the conventional method, even in a larger scale, again showing a good agreement between the slope of point density surface and the phase boundary compositions. Actually, the distance between the phase boundary determined by the conventional method and the maximal slope of point density surface is ~3 at.%, at most, within Figure S3. This value, ~3 at.%, is close to the error range, 3.5%, which is estimated from the statistical analysis mentioned previously (Section 3.1). Thus, it is considered reasonable to regard the maximal slope of point density surfaces from the SDM samples as phase boundary surfaces in multicomponent systems. Taking this route, one could estimate phase boundary surfaces continuously over the entire range of a multicomponent system with high efficiency.

Noting that the equiatomic CrMnFeCoNi composition is located at a point shifted toward the Co/Ni vertex from the center of the tetrahedron, the equiatomic composition is found to be included in region I. Thus, this region is considered to indicate the existence range of the high-entropy solid solution phase. One of the features found regarding region I is that it has a wide compositional range, *x* from 0 to 1, in the Fe*_x_*-(CrMnCoNi)*_1−x_* direction, consistently with the results in the CALPHAD study previously reported [13,27].

The existence of a separate phase, as shown as I′ in Figure 5b, in equilibrium with the high-entropy solid solution phase in the Mn-rich region in the tetrahedron was revealed by the conventional samples at 970 °C, where three-phase equilibrium (I–I′–III) has been confirmed, as seen as an equilibrium triangle in Figure 5b and as micrographical contrast in Figure S4. However, the corresponding region in the slope of the point density map is not clearly separated but connected to the high-entropy solid solution phase. The reason for this could be considered as follows: in this study the compositional data analysis was performed in the Cr–Mn–Fe–Co/Ni tetrahedron space, where the spatial dimension is reduced by one; as seen in Figure 3, the region corresponding to I′ shows a higher Co ratio than that of Ni, and hence, the phase equilibria between the Cantor alloy and the Mn-rich phase could depend on the Co/Ni, ratio resulting in the dull boundaries in the tetrahedron space, which neglects the difference between Co and Ni.

At 800 °C, while it seems as if the existing range of the high-entropy alloys was acquired over its whole compositional range, it is actually partly lacking, especially in the high Mn concentration region, as seen in Figure 5b. This is thought to be due to a larger diffusion distance than that at 970 °C in the corresponding region, since only the composition data from the sample after annealing for 256 h were used at 800 °C, while the data from various annealing conditions from the as-sintered to 256 h annealing samples were compiled at 970 °C. That is to say, the Mn diffusion distance in the sample annealed at 800 °C was too large for its particle size. Thus, in general, it is found that to cover a large compositional range in a targeted multicomponent system, it is important to prepare SDM samples with various diffusion distances relative to the particle size of the elemental powder. To achieve this, the usage of powder with a broad distribution of grain sizes in preparation of the SDM samples or the compilation of data from various annealing times, i.e., diffusion distance, would work, as discussed in Ref. [24].

**Figure 7:**
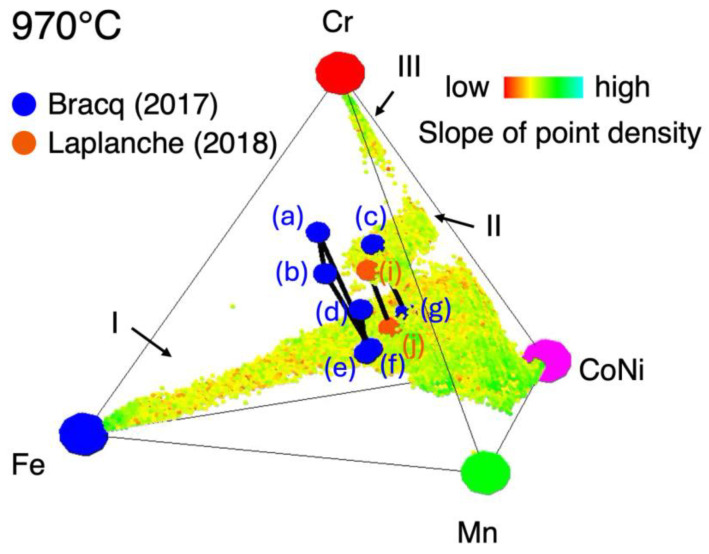
The slope of point density at 970 °C, together with the phase boundary data at 1000 °C in the literature: two-phase equilibrium (i)–(j) [12], (c)–(g), and (d)–(e) [13], and three-phase equilibrium (a)–(b)–(f) [13]. Black segments express tie lines.

Here, the composition range of the high entropy phase (region I) thus determined is compared with those in the literature. Figure 7 shows the slope of point density map at 970 °C in this work, together with the phase boundary data at 1000 °C reported so far in the literature [12,13]. There are four sets of equilibrium compositions: two-phase equilibrium (i)–(j) [12], (c)–(g), and (d)–(e) [13], and three-phase equilibrium (a)–(b)–(f) [13]. Compositions (e), (f), (g), and (j) belong to the FCC high-entropy phase and are found to be located in the vicinity the surface of maximal slope of point density. In addition, points (c) and (i) are from the σ phase and are located in the vicinity of the surface of region II. That again proves that the maximal slope of the point density surfaces can be regarded as phase boundaries. On the other hand, data points were not acquired in this work in the region near the phase boundary data points (a), (b), and (d).

At 800 °C, it is found that the equiatomic composition is included within region I at 800 °C, which means the equiatomic composition is the solid solution phase (region I), in this study. This result is consistent with that in several previous works for equiatomic compositions [6,7,9]. On the other hand, a phase separation occurring at temperatures below 800 °C has been reported for the equiatomic alloy [8]. This might mean that the driving force for the phase separation should be small, even if it occurs.

Figure 6c,f indicate the two-phase microstructures consisting of the Cr phase and an intermetallic phase. The intermetallic phase has been reported as an unknown phase by Keil et al. [28]. The results of this study confirm the presence of the phases in both SDM and the conventional method.

## 4. Conclusions and Remarks

We have applied the sintered diffusion multiple method to the quinary Cr–Mn–Fe–Co–Ni system. The composition ranges of the existing phases in the system have been estimated stereoscopically and continuously in the large part of the entire system at 970 °C and 800 °C, including the high entropy alloy, the so-called Cantor alloy. The validity of the method has been examined, resulting in a good agreement between the results obtained by the sintered diffusion multiple method and those obtained by a conventional method. While data were partially missing due to the too large diffusion rates, depending on composition in the sintered diffusion multiple experiments, this matter could be addressed by using a powder with a wider variety of particle sizes in sample preparation or by annealing the samples for various annealing times.

Here, we note the potential applications of the sintered diffusion multiple method. The results show that this method could be useful in exploring not only the Cr–Mn–Fe–Co–Ni system but also a wide variety of multi-component systems, providing initial insights into their phase diagrams, even of unknown systems. There is a wide variety of alloys and compounds whose properties depend on chemical compositions. Examples include compound semiconductors, intermetallic compounds used as structural materials, magnetic compounds, and so on. The transport properties of compound semiconductors, including thermoelectric materials [29], are sensitive to carrier concentrations, which often depends on chemical compositions. The magnetic properties [30] of compounds often depend on defect structures, which again depend on chemical compositions. Advanced studies on these materials are proceeding to multicomponent systems. The sintered diffusion multiple method can play a critical role in such multicomponent materials.

## Data Availability

The original contributions presented in the study are included in the article/Supplementary Materials, further inquiries can be directed to the corresponding author.

## Supplementary Materials

The following supporting information can be downloaded at: https://www.mdpi.com/article/10.3390/ma18020295/s1, Figure S1: The slope of point density at 970 °C; Figure S2: The slope of point density at 800 °C; Figure S3: Comparison results of the slope of point density surface and the conventional method at 970 °C on cutting section of compositional space of Figure 5b; Figure S4: Microstructure observed in a sample prepared at 970 °C in the conventional method.
