# Supplementary material for: Determination of the Entire Existence Composition Range of CrMnFeCoNi High-Entropy Alloys Using Sintered Diffusion Multiple Method
Source: Materials (Basel). 2025 Jan 10;18(2):295. doi: 10.3390/ma18020295 (PMC11766688; doi:10.3390/ma18020295)
Supplement: Supplementary file 1 [file materials-18-00295-s001.zip › materials-3391451-supplementary.pdf]

Supplementary materials

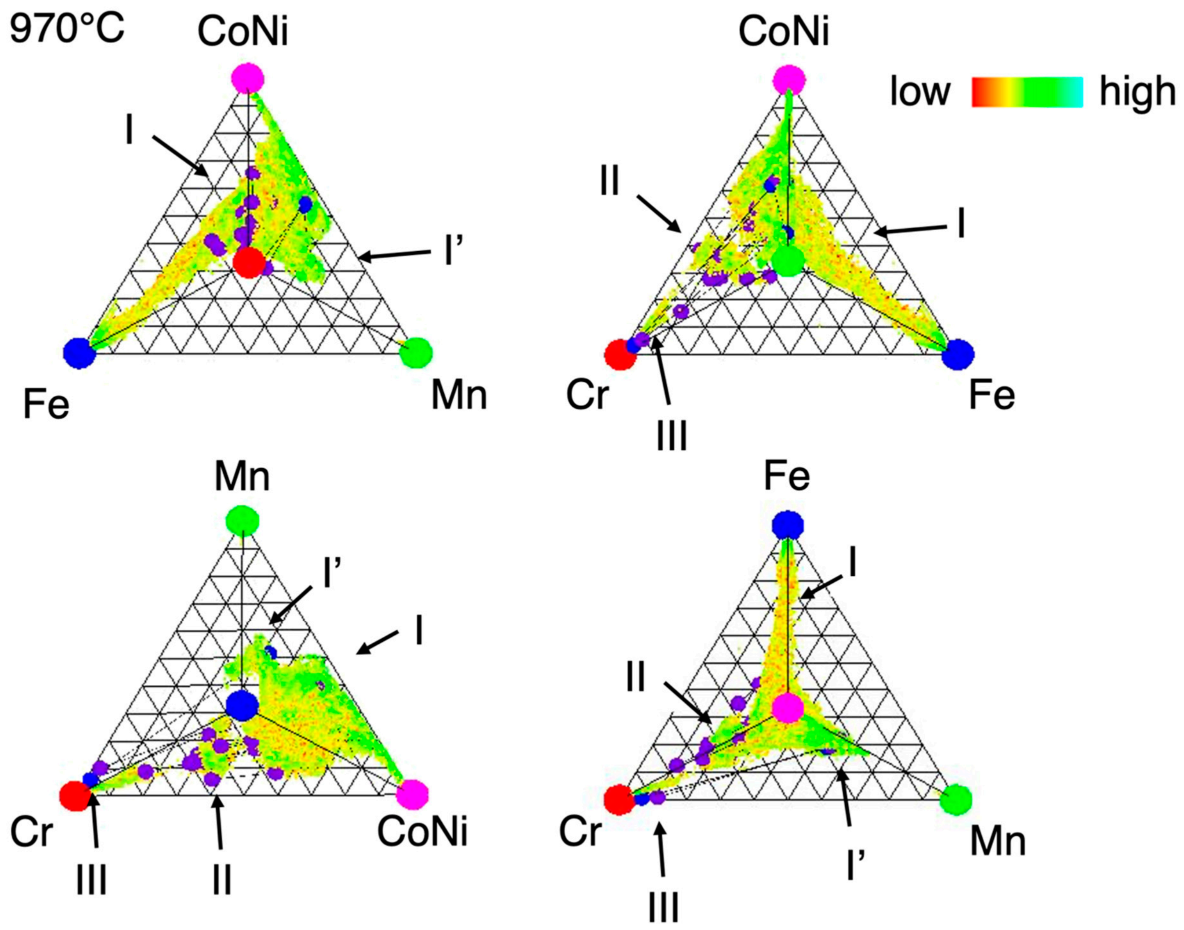

Figure S1. The slope of point density at 970°C. While the data set is the same as that shown in Figure 5 (b), it is seen from various directions different from those of Figure 5 (b).

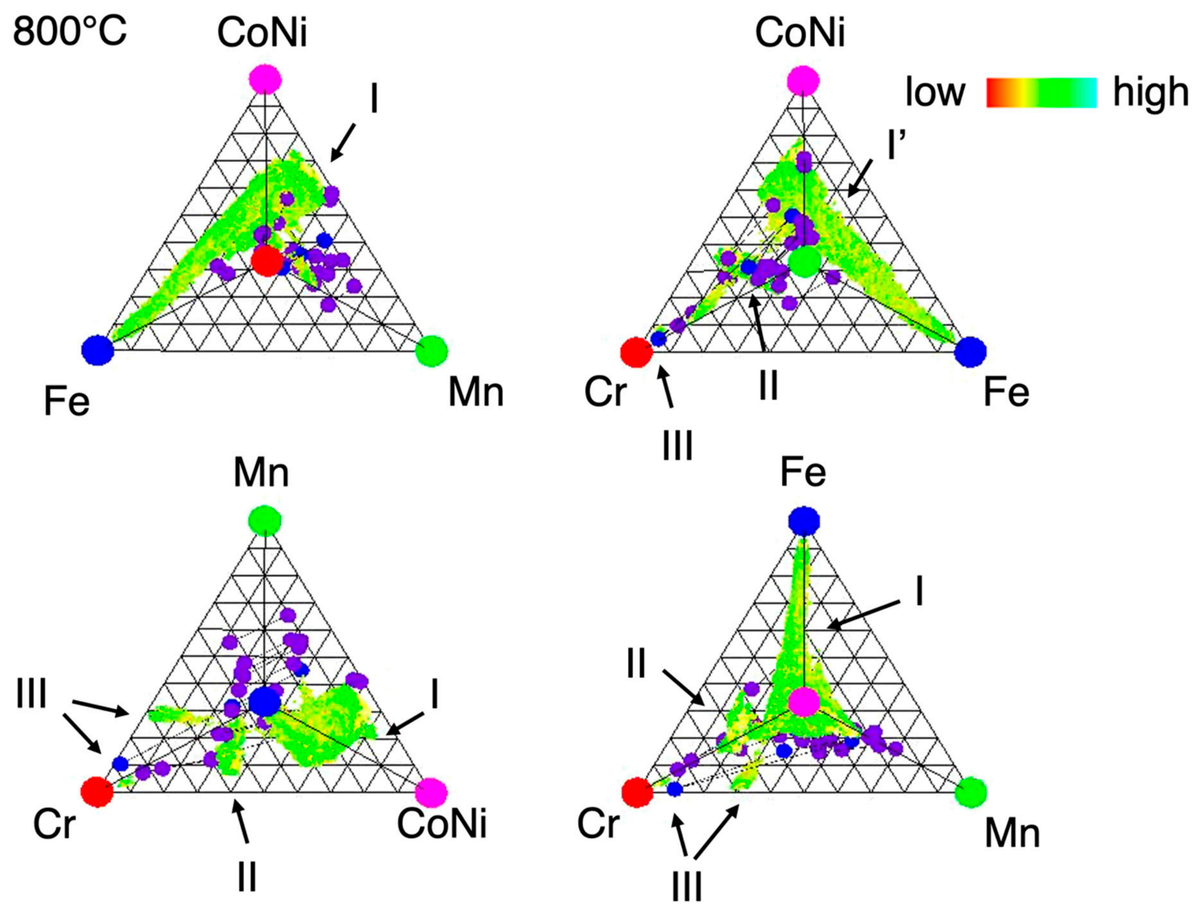

Figure S2. The slope of point density at 800°C. While the data set is the same as that shown in Figure 5 (b), it is seen from various directions different from those of Figure 5 (b).

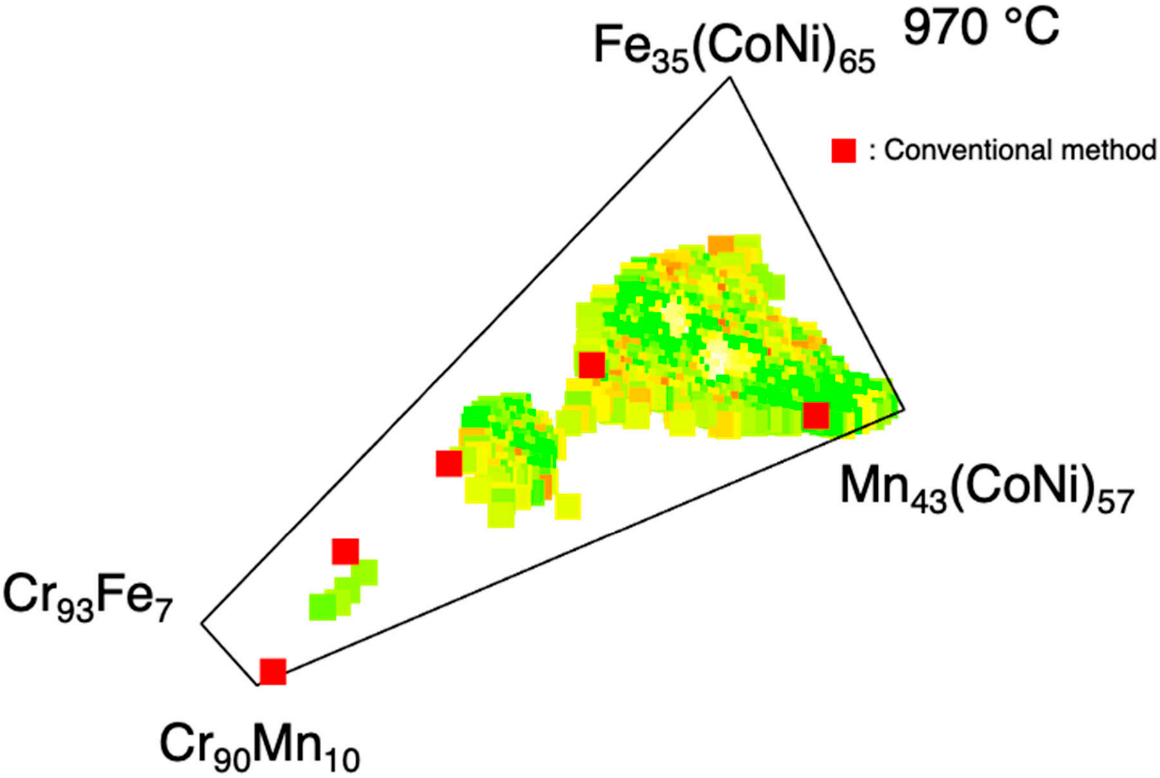

Figure S3. Comparison results of the slope of point density surface and the conventional method at 970 °C on cross section of compositional space of Figure 5 (b). The compositional width in the depth direction of cross section is 1 at. %.

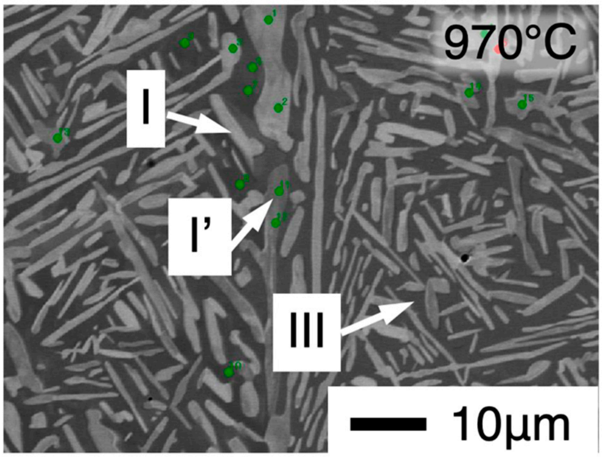

I : $Cr_{12}Mn_{36}Fe_{3}Co_{22}Ni_{27}$

I′: $Cr_{13}Mn_{48}Fe_{11}Co_{16}Ni_{13}$

III: $Cr_{92.6}Mn_{5.8}Fe_{0.2}Co_{0.9}Ni_{0.5}$

Figure S4. Microstructure observed in a sample prepared at 970°C in the conventional method. The compositions shown below the micrograph are those in atomic fractions measured by electron-probe microanalysis for respective phases in the image.
